# P075: Emergence of carbapenem-resistant enterobacteriaceae in surgical and intensive care units of a hospital with low usage of carbapenem in Kano, North West Nigeria

**DOI:** 10.1186/2047-2994-2-S1-P75

**Published:** 2013-06-20

**Authors:** I Yusuf, AH Arzai, MI Getso, A Sherif, M Haruna

**Affiliations:** 1 Microbiology, Bayero University Kano, Nigeria; 2 Microbiology, Bayero University, Kano, Nigeria; 3 Department of Medical Microbiology, Faculty of Medicine, Nigeria; 4 Department of Medical Microbiology, Bayero University Kano, Kano, Nigeria; 5 Department of Biology, Kano state University of Science and Technology, Wudil, Nigeria

## Introduction

Carbapenem Resistant Enterobacteriaceae (CRE) have emerged in surgical (SW) and intensive care units (ICUs) of tertiary health care centers in North West Nigeria despite their low or no usage in the hospitals.

## Objectives

1. To determine the antibacterial susceptibility of pathogens from patients with severe bacterial infections that require rapid and aggressive antimicrobial treatments to imipenem (IMP) and meropenem (MEM) 2. Screen the pathogens for carbapenemase and metallo bêta lactamase production 3. Tests their susceptibility to colistin and tigecycline. 4. To evaluate the views of by different health care practitioners on causes, effects and control of CRE.

## Methods

Isolates from patients admitted for atleast 7 days were screened for susceptibility to IMP and MEM using the CLSI 2012 break points. Carbapenemase and Metallo-β-lactamase (MBL) production were detected phenotypically by Modified Hodge Test (MHT) and Combine Disc Test (CDT) respectively.

## Results

Resistance to IMP and MEM was 10.5% and 12.5% respectively. Carbapenemase production was 34.5% (highest so far in Kano). About 14.4% of the carbapenemase producers produce MBL. All the isolates were susceptible to colistin and tigecycline, except 2 *E. coli* and 1 *K. pneumoniae* which are resistant and also none carbapenemase producers. Of 486 medical practitioners studied in the region, only 1.2% have previous knowledge that MBL causes resistance. About 24.5%, 5.9% and 3.9% have knowledge of ESBL, AmpC and carbapenemase as a cause of rising resistance in the state. In addition, only 38.2% doctors have ever prescribe carbapenems in their professional practices and 12.8% of the doctors only prescribe in a ratio of 1:20 due to its high cost.

## Conclusion

Carbapenem resistance is in increase in hospitals with a low or no usage of carbapenem and the resistances are mediated by carbapenemase especially MBL. Awareness of this problem is low among the medical practitioners that have direct contact with the patients and can contribute to its wide spread in the community.

## Disclosure of interest

None declared.

## References

[B1] CLSI Performance Standards for Antimicrobial Susceptibility Testing 2012 CLSI, Wayne, PA M100 S22

